# Methicillin-resistant Staphylococcus aureus in Nepal

**DOI:** 10.31729/jnma.6251

**Published:** 2021-05-31

**Authors:** Lok Bahadur Shrestha, Gopiram Syangtan, Ajaya Basnet, Krishna Prasad Acharya, Arun Bahadur Chand, Khilasa Pokhrel

**Affiliations:** 1 School of Medical Sciences and The Kirby Institute, University of New South Wales, Sydney, New South Wales, Australia; 2 Tribhuvan University, Shi-Gan International College of Science and Technology, Kathmandu, Nepal; 3 Animal Quarantine Office, Budhanilkantha, Kathmandu, Nepal; 4 Department of Clinical Laboratory, Kist Medical College & Teaching Hospital, Kathmandu, Nepal; 5 Department of Microbiology, Kathmandu Medical College, Sinamangal, Kathmandu, Nepal

**Keywords:** *clindamycin*, *methicillin-resistant Staphylococcus aureus*, *multi-drug resistance*

## Abstract

Staphylococcus aureus is both a frequent commensal and a leading cause of endocarditis, bacteremia, osteomyelitis and skin and soft tissue infections and device-related infections. We performed this minireview to summarize the prevalence of Staphylococcus aureus among clinical samples and estimate the proportion of methicillin-resistant Staphylococcus aureus. The prevalence of Staphylococcus aureus among clinical isolates in Nepal is 34.5%. On average, the proportion of multi-drug resistance in Staphylococcus aureus is 57.1%. Methicillin-resistant Staphylococcus aureus accounts for a total of 41.7%. Inducible clindamycin resistance was detected in about 35% of the isolates. A regular antimicrobial resistance surveillance mechanism is necessary to mitigate the development of resistance among organisms and further spread of superbugs like methicillin-resistance Staphylococcus aureus.

## INTRODUCTION

Staphylococcus aureus is both a frequent commensal and a leading cause of endocarditis, bacteremia, osteomyelitis and skin and soft tissue infections and device-related infections.^1,2^ Methicillin resistance in S. aureus is mediated through an altered protein called low-affinity penicillin-binding protein (PBP2a) which is encoded by the mecA gene which is present in a chromosomal mobile genetic element called Staphylococcal cassette chromosome.^3-5^ The most recent data regarding MRSA incidence obtained from WHO reported values exceeding 20% in all WHO regions, and even 80% in some countries.^6^ MRSA is frequently resistant to most of the commonly used antimicrobial agents including aminoglycosides, macrolides, chloramphenicol, tetracycline, and fluoroquinolones. MRSA strains should be considered to be resistant to all cephalosporins, cephems, and other beta-lactams regardless of the in vitro test results obtained with those agents.^3,4^ We performed this minireview to summarize the prevalence of S. aureus among clinical samples and estimate the proportion of MRSA.

## EPIDEMIOLOGY

The risk of MRSA infection is elevated among children, elderly individuals, athletes, military personnel, individuals who inject drugs, persons with an indigenous background or in urban, underserved areas, individuals with HIV or cystic fibrosis, those with frequent health-care contact, and those in institutionalized populations, including prisoners.^1^ S. aureus colonizes the nares of 28-32% of the world population. Nasal carriage of S. aureus has been recognized as a risk factor for community-acquired and nosocomial infections. MRSA nasal colonization rates range from 0.9% to 1.5%.^7^ Although rates vary by study, colonizing strains genetically match infecting strains in as many as 50-80% of individuals, and MRSA colonization may increase infection risk by as much as 25%. Between the first reports of MRSA in 1961 and the 1990s, the infection was generally associated with healthcare contact. There are two distinct types of MRSA: hospital-acquired MRSA (HA-MRSA) and community-acquired MRSA (CA-MRSA). CA-MRSA originated with individuals in the community who had no risk factors from exposure to the hospital environment and had distinctly different antibiotic sensitivities than the HA-sMRSA which infected hospitalized patients with specific risks of infections. By the 1990s, cases of MRSA infection emerged in individuals who had no prior hospitalization, leading to separate definitions for HA-MRSA and CA-MRSA.^1^

## STAPHYLOCOCCUS AUREUS IN NEPAL

Staphylococcus aureus, a notorious human pathogen, is a major cause of the community as well as healthcare-associated infections. It can cause a diversity of recalcitrant infections mainly due to the acquisition of resistance to multiple drugs, its diverse range of virulence factors, and the ability to produce biofilm in indwelling medical devices. We obtained a total of 14,647 organisms in these research articles, among which 5,338 (34.45%, 95% CI 35.66%-37.24%) were Staphylococcus aureus. The proportion of S. aureus among the total organisms isolated ranged from 6.64%^8^ to 100%.^9^ The most common source of S. aureus is pus sample^10,11^ while some studies also suggested that S. aureus is also commonly isolated from sterile body fluids^12^ and urine specimen.^13-15^ A study conducted in Palpa reported a total of 133 S. aureus isolates from 1981 samples (blood 647, pus 188, swab 321, body fluid 354, and urine 471). The maximum number of S. aureus was found in children <10 years (49.1%) and a higher incidence of MRSA infections was found in males (52.4%).^16^ A study was conducted in Kathmandu among 50 health care workers (HCWs), 92% (n=46) were identified as S. aureus carriers in nose or hand or both with 58% being MRSA carriers. Thirty-six percent of the participants had S. aureus and 16% had MRSA on their hands. Similarly, S. aureus was detected in 90% and MRSA in 54% in the nares of HCWs.^17^ A similar type of research was conducted in Bhairawaha, where, out of 204 HCWs, 32 (15.7f%) were nasal carriers of S. aureus and among them, 7 (21.9%) was the carrier of MRSA. Nasal carriage among male and female HCWs were 19.4% (21/108) and 11.5% (11/96) respectively.^18^ Another study conducted in Kathmandu reported 27.13% (35/129). HCWs were identified as nasal carriers of S. aureus. The nasal carriage was at least 21.73% among doctors and was highest 43.47% among ward attendants and was 30% among nursing students and 22.22% among nursing staff.^19^

## METHICILLIN-RESISTANT STAPHYLOCOCCUS AUREUS

The evidence suggests that the populations harbouring S. aureus and its methicillin-resistant (MRSA) strains are at higher risk for developing an invasive infection.^1,2^ The overall proportion of MRSA is found to be 41.7% (2229/ 5338) in Nepal. The prevalence of MRSA varied greatly among different studies ranging from 14.64%^13^ to 81.64%.^20^ A study conducted in Chitwan regarding nasal colonization among 200 participants concluded that S. aureus was present in 15% of individuals with 26.7% (n=8) of them being MRSA and 4 (13.3%) isolates were found to have inducible clindamycin resistance by D-zone test.^21^ Another study in the sample hospital reported 43.1% of the isolates were resistant to methicillin by the cefoxitin method, and 39.2% were resistant to oxacillin. Of the 100 isolates exhibiting erythromycin resistance, 38 (12.4%) were found to have inducible clindamycin resistance.^22^ A similar type of research was conducted in Kathmandu, Nepal^20^ which concluded that among 161 S. aureus isolates, 131 (81.4%) isolates were identified as MRSA and the remaining 30 (18.6%) isolates as MSSA strains. The MRSA isolates were significantly more resistant to the majority of the antibiotics than the MSSA strains. Similarly, a study conducted at Dharan among urinary tract infection reported that 44% (7/18) S. aureus were methicillin-resistant.^15^ The study conducted in Bhairawaha concluded that the overall nasal carriage rate of MRSA was 3.4% (7/204). S. aureus carriage rate was highest among doctors 20.8% (15/72) while MRSA carriage rate was highest among nurses 7.8% (4/51).^18^ In a study conducted in Pokhara, 139 MRSA isolates from various clinical specimens were included. Out of these, 35.2% (49/139) were HA-MRSA, 59.7% (83/139) were CA-MRSA and 5% (7/139) were from hospital environment.^23^ Methicillin-resistance is also an emerging problem in coagulase-negative Staphylococci (CoNS). Several studies conducted in Nepal points out a high prevalence of MR-CoNS.^24,25^

## MULTI-DRUG RESISTANCE

Multi-drug resistance is defined as resistance to at least one antimicrobial agent in three or more classes of antibiotics were determined in some studies.^26^ On average, the proportion of MDR among S. aureus isolates was found to be 55.17%. However, we noticed a wide range of prevalence in isolation of MDR S. aureus ranging from 24.3^27^ to 86%.^13^ In a study conducted by Chitwan,^28^ 60.5% of the total isolates, were MDR; interestingly, out of 30 biofilm-positive isolates, 26 (86.7%) were MDR, whereas 4 (13.3%) were non-MDR. In contrast, no MDR isolates were noted among any of the biofilm nonproducers; in other words, all the biofilm non-producers were non-MDR isolates (P<0.05). A study conducted in Dharan reported multi-drug resistance in 71.4% (10/14) isolates obtained from catheter-associated urinary tract infection, while only 30.4% of isolates obtained from community-acquired UTI were multi-drug resistant.^15^ Another study conducted in Kathmandu^8^ showed 78.8% of S. aureus isolates were MDR, while research from Pokhara^29^ reported 29.5% (12/44) of S. aureus were multidrug-resistant and 14 31.8% were biofilm producers. Similarly, a study conducted in Lalitpur^30^ revealed 27.7% MDR (45/112) among S. aureus isolates. MDR was higher in pus samples (66.6%) as compared to blood (4.6%) and body fluids (0%). A similar study conducted in Kathmandu reported that 49.5% of Staphylococcus aureus were MDR and 31.56% isolates were methicillin resistant.^31^

## INDUCIBLE CLINDAMYCIN RESISTANCE (ICR)

A study conducted by Kathmandu^20^ reported constitutive MLSB phenotype in 10.5% (n=17), inducible MLSB phenotype in 34.8% (n=56), macrolide sensitive (MS) phenotype 35.5% (n=57). When they compared the results statistically, the constitutive MLSB phenotype was determined to be 7.5 times greater (P=0.001, OR 9.9, 95% CI 2.5-39.2) and the inducible phenotype 3 times greater (P=0.361, OR 2.4, 95% CI 0.367-15.7) in MRSA than MSSA isolates. Another study conducted in Lalitpur^10^ concluded that inducible macrolide-lincosamide-streptogramin B (MLSB) resistance, constitutive MLSB resistance, and macrolide-streptogramin B (MSB) resistance were seen in 17 (22.4%), 8 (10.5%), and 17 (22.4%) S. aureus respectively. Inducible MLSB resistance was higher among MRSA in comparison to MSSA (p<0.05). A similar study conducted in Kathmandu^32^ revealed that inducible clindamycin resistance was observed in 11 isolates, among which, 6 were MSSA and 5 were MRSA. Statistically, there was no significant association between methicillin resistance and inducible clindamycin resistance (p value>0.05). A study conducted in Dharan demonstrated that among 300 S. aureus 41% were methicillin-resistant and MRSA demonstrated 11.6% constitutive MLSBc. D test positive inducible resistance (MLSBi) was found to be 24.59% and (22.4%) were MS type among MRSA isolates.^33^ A study conducted in Chitwan found a 13.3% prevalence of ICR among S. aureus isolates.^21^ A research is done in Lumbini Medical College and Teaching Hospital reported MLSBi, MLSBc, and sensitive phenotype in 25.6%, 22.6%, and 51.9% of the total respectively. A higher number of MLSB resistant organisms (40/133; 62.5%) were resistant to methicillin (p< 0.001).^16^ Similar study conducted in Kathmandu observed methicillin resistance and inducible clindamycin resistance (iMLSB) were observed in 31.4% and 10% of Staphylococcus aureus isolates respectively.^34^ A research conducted in Kathmandu observed inducible MLSB-(iMLSB) resistance, constitutive MLSB resistance, and macrolide-streptogramin B (MSB) resistance were detected in 30 (24%), 19 (15.2%), and 34 (27.2%) isolates of S. aureus, respectively.^35^

## MECA GENE DETECTION

Methicillin resistance in S. aureus is mediated through an altered protein called low-affinity penicillin-binding protein. PBP2a is encoded by mecA gene which is present in a chromosomal mobile genetic element called Staphylococcal cassette chromosome mec. A study conducted by Adhikari, et al.^32^ in Kathmandu showed that a total of 32 (29.1%) S. aureus isolates contained mecA gene. All of the mecA containing strains of S. aureus were MRSA by both phenotypic methods. A similar study conducted in Dharan concluded that mecA gene was detected in 71.1% of the total Staphylococcus isolates.^5,36^ Another research conducted in Pokhara reported mecA and PVL genes detection in 79/139 (56.8%) of the isolates. The majority of the PVL positive isolates were obtained from pus samples accounting for 74/98 (75.5%).^23^
